# Hippocampal Sector–Specific Metabolic Profiles Reflect Endogenous Strategy for Ischemia-Reperfusion Insult Resistance

**DOI:** 10.1007/s12035-020-02208-6

**Published:** 2020-11-22

**Authors:** Olga Krupska, Tomasz Kowalczyk, Małgorzata Beręsewicz-Haller, Paulina Samczuk, Karolina Pietrowska, Krzysztof Zabłocki, Adam Kretowski, Michal Ciborowski, Barbara Zabłocka

**Affiliations:** 1 Molecular Biology Unit, Mossakowski Medical Research Centre PAS, Warsaw, Poland; 2 Laboratory of Cellular Metabolism, Nencki Institute of Experimental Biology, Warsaw, Poland; 3 Metabolomics Laboratory, Clinical Research Centre, Medical University of Bialystok, Białystok, Poland

**Keywords:** *Meriones unguiculatus*, Transient ischemic episode, Ischemia-reperfusion injury, Hippocampus, CA1, *Gyrus dentatus*, Untargeted metabolomics, LC-QTOF-MS

## Abstract

The gerbil is a well-known model for studying cerebral ischemia. The CA1 of the hippocampus is vulnerable to 5 min of ischemia, while the CA2–4 and dentate gyrus (DG) are resistant to it. Short-lasting ischemia, a model of transient ischemic attacks in men, results in CA1 neuron death within 2–4 days of reperfusion. Untargeted metabolomics, using LC-QTOF-MS, was used to enrich the knowledge about intrinsic vulnerability and resistance of hippocampal regions and their early post-ischemic response (IR). In total, 30 significant metabolites were detected. In controls, taurine was significantly lower and guanosine monophosphate was higher in CA1, as compared to that in CA2–4,DG. LysoPG and LysoPE were more abundant in CA1, while LysoPI 18:0 was detected only in CA2–4,DG. After IR, a substantial decrease in the citric acid level in CA1, an accumulation of pipecolic acid in both regions, and opposite changes in the amount of PE and LysoPE were observed. The following metabolic pathways were identified as being differentially active in control CA1 vs. CA2–4,DG: metabolism of taurine and hypotaurine, glycerophospholipid, and purine. These results may indicate that a regulation of cell volume, altered structure of cell membranes, and energy metabolism differentiate hippocampal regions. Early post-ischemia, spatial differences in the metabolism of aminoacyl-tRNA biosynthesis, and amino acids and their metabolites with a predominance of those which upkeep their well-being in CA2–4,DG are shown. Presented results are consistent with genetic, morphological, and functional data, which may be useful in further study on endogenous mechanisms of neuroprotection and search for new targets for therapeutic interventions.

**Supplementary Information:**

The online version contains supplementary material available at 10.1007/s12035-020-02208-6.

## Introduction

Cerebral stroke is a significant clinical problem. In the next decades, in an aging society, the number of strokes as well as transient ischemic attacks (TIA) will increase. TIA is defined as a transient episode of neurologic dysfunction due to the focal or global brain injury. Currently, the number of TIA incidence in the USA could be around half a million per year, and estimates are about 1.1 per 1000 in the US population [1]. At present, no neuroprotective agents have been shown to impact the clinical outcomes in cerebral stroke and TIA cases, but new compounds and therapies are still emerging [2]. In this study, we have focused on the global transient ischemia that reflects a short-lasting impairment of the brain supplying with oxygen and fuel (i.e., due to heart attack or drowning) rather than brain blood vessel occlusion or hemorrhage.

Neuronal injury after transient global ischemia is induced by a common action of hypoxia, hypoglycemia, and glutamate excitotoxicity. Post-ischemic neuronal death develops gradually upon reperfusion and selectively damages neurons in specific brain areas due to their intrinsic selective vulnerability. In rodent models, ischemia-reperfusion injury (IR) typically affects neurons in the hippocampal CA1 area while neurons in the CA2-CA3-CA4 and granule cells of the dentate gyrus (DG) are relatively resistant [3, 4]. Molecular mechanisms underlying the differential vulnerability of hippocampal areas (dorsal CA1 vs. abdominal CA2–4,DG) have evoked great curiosity.

Gerbil (*Meriones unguiculatus*) belong to Rodentia; however, in contrast to other members of this family, it displays many unique features more similar to those in humans, including vision, audition, and sensitivity to the low sound frequency range as well as genome sequence ([5] and references therein). Therefore, gerbils are well suited to study a range of pathophysiological conditions, including seizures and cerebral ischemia. Transient bilateral common carotid artery occlusion for several minutes induces global cerebral ischemia, due to an incomplete circle of Willis, resulting in delayed neuronal cell death in the CA1 [3]. Short-lasting global brain ischemia in gerbils is a model of transient ischemic attacks in humans, leading to serious cognitive impairment, especially in older people.

Post-ischemic neuronal cell death was first and foremost associated with glutamate neurotoxicity [6, 7] as well as seriously impaired cellular energy metabolism and reactive oxygen species handling [8, 9], calcium signaling [10], lipid metabolism and its role in signal transduction [11, 12], and purine metabolism [13, 14], to mention only the most important research threads. Multiple differences between pyramidal cells in CA1 and CA3 have been described with the use of anatomical, electrophysiological, molecular, and genetic techniques [15, 16] seeking for the cause of selective vulnerability of CA1. On these days, the search for molecular differences of regions of the hippocampus exhibiting the opposite outcome after short brain ischemia is the subject of many studies [17–19]. Recently, proteomic and transcriptomic studies paved a new way of looking for a cause of neurons’ delayed death or survival after IR episodes and for candidates for ischemic brain treatment [20]. Moreover, metabolomics was applied to investigate cerebral injury and protection in mice and rat models of brain ischemia [21–25]. Research has been carried out using various metabolomic techniques and in various models of global ischemia and stroke. As a result of global approach, biomarkers in cerebral ischemia are listed [24]. Regardless of the research model and experimental approach used, metabolism of amino acids and lipids, glycolysis, and mitochondrial energy conversion is indicated as the most changed. Metabolomics approach has also been used to search for biomarkers of stroke severity in patients’ sera [26].

Since none of abovementioned data fully explains the intrinsic selective vulnerability and resistance to ischemic episode in vivo and in vitro, the present study was devoted to determine the metabolites differentiating CA1 and CA2–4,DG in control brain prior to any treatment. Moreover, on the basis of previously established time course of the post-ischemic processes which occur in hippocampal sectors [27–29], 1-h-lasting reperfusion time was selected to look for changes in metabolites that can be engaged in endogenous neuroprotection. Previously observed rapid post-ischemic protein activation and translocation to mitochondria [30, 31] suggest possible changes of cell metabolism and metabolite content much earlier than neuronal damage in CA1 visible 2–4 days after reperfusion [32]. The process of glial scar formation is accomplished 6–7 days post-ischemia [3].

Here we have shown that metabolic fingerprints of control and early post-ischemic gerbil hippocampal regions (vulnerable and resistant to a transient ischemic episode) differ. These results indicate differences in the regional metabolism within hippocampi not exposed to ischemic treatment and their additional modifications due to IR episode.

## Materials and Methods

### Animals

Three-month-old male gerbils (*Meriones unguiculatus*) were obtained from the Animal House of Mossakowski Medical Research Centre, PAS (Warsaw, Poland). All procedures involving animals were approved by the I Local Ethics Committee for Animal Experimentation (permission number 379/2017). All efforts were made to minimize animal suffering and to reduce the number of specimens used.

### Gerbil Model of Transient Global Brain Ischemia

**Fig. 1:**
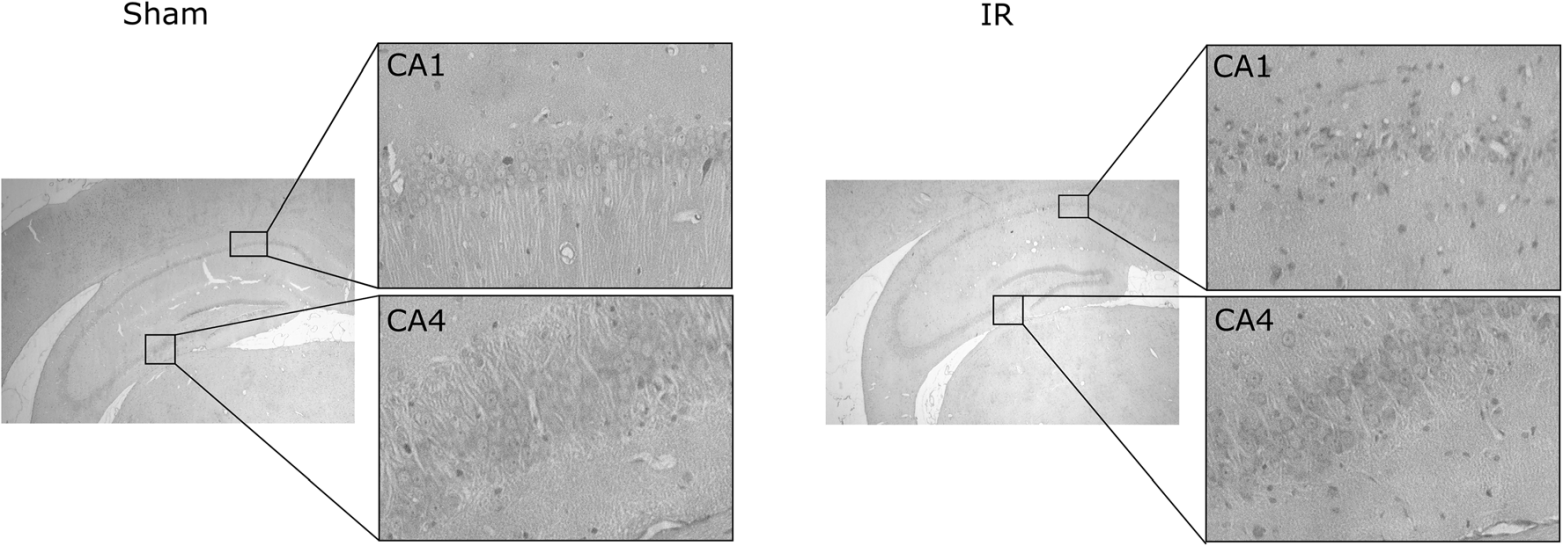
Transient brain ischemia and reperfusion causes neurodegeneration in the CA1 hippocampus. Brain sections of control and after 5 min of ischemia followed by 7 days of recovery, animals were stained with hematoxylin/eosin and photographed under a light microscope with 2.5 or 20 magnification

Male gerbils weighing 50–60 g were used. The ischemic insult was performed by 5-min ligation of the common carotid arteries under isoflurane in O_2_ anesthesia in strictly controlled normothermic conditions, as described previously [33]. Sham animals, which underwent the same surgical procedure, but without the actual ligation, served as controls. Groups of sham operated and ischemic gerbils (3 animals in each group) were allowed for recovery periods of 1 week; then, for histological examinations, they were perfused with ice-cold 4% paraformaldehyde in PBS under ketamine-xylazine anesthesia. The histological evaluation was performed on paraffin-embedded, 10-μm-thick sections stained with hematoxylin/eosin. The morphology of hippocampi and extent of cell damage of the CA1 region were visualized using a Zeiss Axioscope 2 bright-field microscope (Fig. 1). Two other groups of sham and ischemic animals (3 gerbils in each group) were decapitated 1 h after the surgery. Hippocampi were quickly isolated from both hemispheres and divided into two regions, i.e., CA1 and CA2–4,DG, as it was reported previously [34]. Manual “unfolding” of the dorsal hippocampus, containing CA1 sector, was completed under binocular with a *fissure hippocampalis* taken as a starting orientation point. Consequently, four different types of samples were obtained: control CA1 (C_CA1), control CA2–4,DG (C_CA2–4,DG), ischemia injury CA1 (IR_CA1), and ischemia injury CA2–4,DG (IR_CA2–4,DG). All the samples were snap-frozen in liquid nitrogen and stored at − 80 °C until further processing.

### Chemicals and Reagents

Purified water was obtained using the Milli-Q Integral 3 system (Millipore SAS, Molsheim, France). Zomepirac sodium salt (used as internal standard, IS), LS-MS-grade acetonitrile (ACN), methanol (MeOH), and formic acid (FA) were purchased from Sigma-Aldrich Chemie GmbH (Steinheim, Germany). Pure p.a. ammonium solution (25%) was purchased from Avantor Performance Materials (Gliwice, Poland). The API-TOF reference mass solution kit (G1969-850001) and tuning solutions, ESI-L low concentration tuning mix (G1969-85000), and ESI-TOF Biopolymer Analysis reference masses (G1969-850003) were purchased from Agilent Technologies (Santa Clara, CA, USA). Standards to confirm the identity of metabolites were purchased from Sigma-Aldrich Chemie GmbH (Steinheim, Germany) (taurine, pipecolic acid, leucine, histidine, phenylalanine, 3-methylhistidine, citric acid, tryptophan, adenosine, and stearoylcarnitine) and Avanti Polar Lipids Inc. (AL, USA) (LPI 18:0 and LPG 18:1).

### Sample Treatment

Sample treatment was performed using the previously described LC-MS methods [35]. Brain tissue samples (10 mg) were placed in Eppendorf tubes together with two steel beads (5 mm) and 200 μL of freeze cold (− 20 °C) 50% methanol. Samples were homogenized using a bead mill homogenizer (TissueLyser LT; Qiagen Hilden, Germany) for 8 min (30 Hz). After homogenization, beads were removed and 200 μL of freeze cold (− 20 °C) acetonitrile containing 1 ppm of zomepirac was added to the samples. Metabolites were extracted by vortex-mixing of the samples for 1 h. After extraction, samples were centrifuged (Eppendorf, Hamburg, Germany) at 21,000×*g* for 20 min at 20 °C. The supernatant was filtered through a 0.22-μm nylon filter (Thermo Fisher Scientific, Waltham, MA, USA), and an equal volume of each sample was pooled to get a quality control (QC) sample. The remaining volume was divided into two parts: one for the LC-RP-MS analysis and second (diluted 1:1 with acetonitrile) for LC-HILIC-MS analysis. Blank extraction (prepared following the exact same procedure as biological samples but not containing tissue) was also prepared and analyzed together with biological samples.

### Metabolic Fingerprinting

Samples were randomly analyzed by an LC-MS system consisted of 1290 Infinity UHPLC with a degasser, two binary pumps, and a thermostated autosampler coupled to a 6550 iFunnel Q-TOF-MS detector (both Agilent Technologies, Santa Clara, CA, USA). Analyses were performed in positive (ESI+) and negative (ESI−) ion modes.

LC-RP-MS analysis was performed, whereby 1 μL of the sample was injected into a thermostated (60 °C) Zorbax Eclipse Plus C8 RRHD (2.1 × 150 mm, 1.8 μm particle size, Agilent Technologies) chromatographic column. The flow rate was 0.6 mL/min with solvent A (water with 0.1% formic acid) and solvent B (acetonitrile with 0.1% formic acid). The chromatographic gradient started at 25% of phase B and was increasing to reach 95% of phase B in 14 min. This proportion was kept for 1 min, and after that, the gradient returned to initial conditions (25% of phase B) in 0.1 min and was maintained at this solvent proportion for 4.9 min in order to re-equilibrate the system for the next injection. The mass spectrometer was operated in full scan mode from the mass (*m*/*z*) 50–1000. The capillary voltage was set to 3 kV for positive and 4 kV for negative ionization modes; nozzle voltage was 1000 V; the drying gas flow rate was 12 L/min at 250 °C and gas nebulizer at 52 psig; the fragmentor voltage was 250 V for positive and negative ionization modes. Data were collected in centroid mode at a scan rate of 1.5 scans per second. Accurate mass measurements were obtained by means of calibrant solution delivery using a dual-nebulizer ESI source. A calibrating solution containing reference masses at *m*/*z* 121.0509 (protonated purine) and *m*/*z* 922.0098 (protonated hexakis (1H,1H,3H-tetrafluoropropoxy) phosphazine or HP-921) in positive ion mode or *m*/*z* 119.0363 (proton abstracted purine) and *m*/z 966.0007 (formate adduct of HP-921) in negative ion mode was continuously introduced by an isocratic pump (Agilent, Santa Clara, CA, USA) at a flow rate of 0.75 mL/min (1:100 split).

To perform LC-HILIC-MS analysis, the extracted sample was diluted twice with acetonitrile, and 0.5 μL of the diluted sample was injected into a thermostated (30 °C) Poroshell Hilic 2.1 × 100 mm, 2.7-μm column (Agilent Technologies). The flow rate was 0.1 mL/min with solvent A (10 mM ammonium formate in water, pH = 4) and solvent B (acetonitrile with 0.1% formic acid). The gradient started at 70% of phase B and was decreasing to reach 60% of phase B in 8 min. Subsequently, to clean the column, the gradient was increasing to 95% of phase B in 0.1 min, and this proportion was kept for 0.8 min. After that, the gradient returned to initial conditions (70% of phase B) in 0.1 min and was maintained at this solvent proportion for 3 min in order to re-equilibrate the system for the next injection. The mass spectrometer was operated in full scan mode from m/z 50–350. The capillary voltage was set to 3.5 kV for both ESI modes; the drying gas flow rate was 13 L/min at 200 °C and gas nebulizer at 30 psig; the fragmentor voltage was 200 V for both ESI modes. Measured *m*/*z* values were corrected with the use of two reference masses at *m*/*z* 121.0509 (protonated purine) and *m*/*z* 322.0481 (protonated hexamethoxyphosphazine or HP-0321) in positive ion mode or *m*/*z* 112.9855 (TFA anion) and *m*/*z* 301.9981 (tris(2,4,6-trifluoromethyl)-1,3,5-triazine or HP-0285) in negative ion mode, which were continuously introduced by an isocratic pump (Agilent, Santa Clara, CA, USA) at a flow rate of 0.8 mL/min or 1 mL/min (1:100 split) in ESI+ or ESI− modes, respectively.

### Measurement of the Protein Content

The protein pellet which remained after the extraction of metabolites was re-suspended in 150 μL of radioimmunoprecipitation assay buffer (RIPA buffer) and subjected to sonication in a water bath at 60 °C for 30 min. After that, samples were centrifuged for 15 min at 15,000×*g*. The protein concentration was measured using a BCA kit (Pierce BCA protein assay kit, Thermo Scientific) according to the manufacturer protocol.

### LC-MS Data Treatment

The raw data collected by the analytical instrumentation were cleaned of background noise and unrelated ions by the molecular feature extraction (MFE) tool in the Mass Hunter Qualitative Analysis Software (B.07.00, Agilent, Santa Clara, CA, USA). The MFE creates a list of all possible components described by mass, retention time (RT), and abundance. The limit for the background noise for data extraction by MFE was set to 500 and 1000 counts for positive and negative ion modes in the LC-HILIC-MS analysis and 1200 counts for both ion modes in the LC-RP-MS analysis. To identify co-eluting adducts of the same feature, the following adduct settings were applied: +H, +Na, +K in positive ion mode and −H, +HCOO, +Cl for negative ion mode. Dehydration neutral losses were also allowed in both ionization modes. Additionally, +NH4 was included in the list of possible adducts for data recorded in HILIC ESI+ mode. Sample alignment and data filtering were performed using Mass Profiler Professional 12.6.1 (Agilent, Santa Clara, CA, USA). Parameters applied for the alignment were 1% for RT and 15 ppm for the mass variation. In the first step of the data treatment, signals from the blank analysis were removed. In the quality assurance (QA) procedure, metabolic features detected in > 75% in QC samples with the coefficient of variation (CV) < 30% were kept for further data treatment. Data were also filtered to keep metabolic features present in 100% of samples in at least 1 out of 4 groups. Before statistical analysis, for each pair of comparisons, additional data filtering was performed. For each metabolic feature, only one missing value in each of the compared groups was allowed. However, metabolic features with only one missing value in one group and all missing values in the other group were also kept. Obtained data were normalized to protein amount in the sample.

### Statistical Analysis

Statistical analysis was performed to find metabolites differentiating brain tissue from gerbil’s with and without ischemic episodes as well as to find metabolites discriminating CA1 and CA2–4,DG hippocampus areas. A multivariate statistic was used to select metabolites which contribute the most into the groups’ separation. A partial least squares discriminant analysis (PLS-DA) was applied to log-transformed data. The following comparisons were performed: C_CA1 vs. C_CA2–4,DG; IR_CA1 vs. IR_CA2–4,DG; IR_CA1 vs*.* C_CA1; IR_CA2–4,DG vs. C_CA2–4,DG. The comparisons were performed independently for each data set. The contribution of metabolite to sample discrimination observed on obtained plots was assessed based on the volcano plots created by plotting variable importance in the projection (VIP) against loading values scaled as correlation coefficient values [*p*(corr)] generated based on the obtained PLS-DA models. Variables with VIP > 1.0 and absolute *p*(corr) > 0.4 were considered significant. Multivariate statistics and plots were performed using SIMCA−P + 13.0.3.0 (Umetrics, Umea, Sweden).

### Metabolite Identification

The identification of metabolites was performed based on the MS/MS fragmentation, as previously described [36]. Accurate masses of features were searched against the METLIN, KEGG, LIPIDMAPS, and HMDB databases, which were simultaneously accessed by CEU Mass Mediator (available on-line search engine, http://ceumass.eps.uspceu.es/mediator/). The identity of metabolites was confirmed by matching the experimental MS/MS spectra to MS/MS spectra from databases or to fragmentation spectra and retention time obtained for the metabolite’s standard (if available). Experiments were repeated with identical chromatographic conditions to the primary analysis. Ions were targeted for collision-induced dissociation (CID) fragmentation on the fly based on the previously determined accurate mass and retention time. Characteristic fragments of identified metabolites together with retention time, an error of mass measurement, adduct formed, and ionization mode and chromatography in which metabolite was detected are presented in Supplementary Table S1.

### MetaboAnalyst 4.0 Analysis

The pathway analysis was performed with MetaboAnalyst 4.0 (http://www.metaboanalyst.ca/). This on-line tool allows analyzing the impact of particular compounds on biochemical pathways specifically for metabolomics studies. In version 4.0, there are currently 15 pathway libraries supported, with a total of 1173 pathways. The pathway analysis module combines results from the powerful pathway enrichment analysis with results from the pathway topology analysis. Pathway analysis accepts a list of compound labels (common names, HMDB IDs, or KEGG IDs). Next, Fisher’s exact test or hypergeometric test is used. The results from the pathway analysis can be presented graphically as well as in the form of table [37].

## Results

### Metabolomics of the Gerbil Hippocampus

**Fig. 2:**
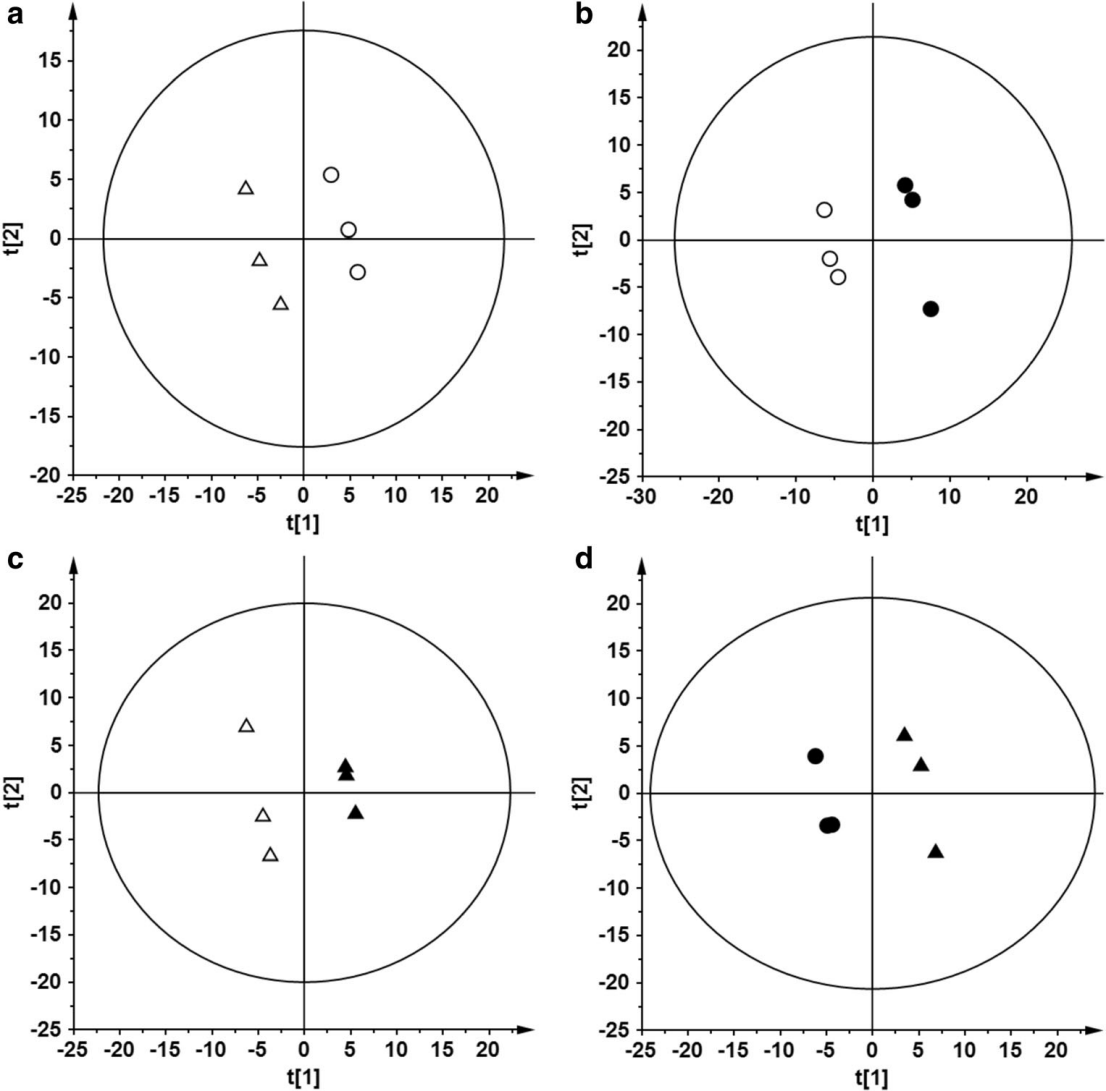
Partial least squares discriminant analysis plots showing discrimination between the studied hippocampal regions. Plots were obtained based on the RP(+) data, which were log-transformed and Par scaled. White circle, C_CA1; white up-pointing triangle, C_CA2–4,DG; black circle, IR_CA1; black up-pointing triangle, IR_CA2–4,DG. Panel **a**: C_CA1 vs. C_CA2–4,DG; *R*^2^ = 0.996, *Q*^2^ = 0.677. Panel **b**: C_CA1 vs. IR_CA1; *R*^2^ = 0.998, *Q*^2^ = 0.794. Panel **c**: C_CA2–4,DG vs. IR_CA2–4,DG; *R*^2^ = 1.000, *Q*^2^ = 0.890. Panel **d**: IR_CA1 vs. IR_CA2–4,DG; *R*^2^ = 0.996, *Q*^2^ = 0.765

Following 5 min of ischemia, all the animals developed typical hippocampal damage observed morphologically after 7 days of reperfusion. The lesion was present throughout the extent of the dorsal hippocampus, whereas damage outside the CA1 hippocampus was not observed (Fig. 1). Brain tissue samples obtained from the gerbil hippocampus (CA1 and CA2–4,DG) without and with ischemic episodes followed by 1 h of reperfusion were fingerprinted using LC-QTOF-MS. Samples were analyzed in two ion modes (ESI+ and ESI−), with the use of two types of chromatography: reversed-phase liquid chromatography (RP) and hydrophilic interaction liquid chromatography (HILIC). Consequently, four data sets were obtained: HILIC(+), HILIC(−), RP(+), and RP(−). Each data set was analyzed independently. After quality assurance procedures, 146, 84, 209, and 173 metabolic features remained for HILIC(+), HILIC(−), RP(+), and RP(−) data, respectively. For each data set, PCA modeling was performed to locate the quality control (QC) samples. Close clustering of the QC samples observed on PCA plots (Supplementary Figure S1) indicates the proper quality of the data. To check the samples’ classification, a PLS-DA model was built for each data set. For each comparison, PLS-DA plots obtained from RP(+) data are presented in Fig. 2. The rest of the models are presented in Supplementary Figures S2–S4. To indicate significant metabolic features, statistical analysis (as described in the “Materials and Methods” section) was performed, giving 52, 70, 69, and 69 significant metabolites for each comparison: C_CA1 vs*.* C_CA2–,DG, IR_CA1 vs. IR_CA2–4,DG, IR_CA1 vs. C_CA1, and IR_CA2–4,DG vs*.* C_CA2–4,DG, respectively. Among significant metabolic features, 30 key markers that contributed to group separation between hippocampal sectors in control and after IR episodes have been identified. A comparison of CA1 and abdominal (CA2–4,DG) parts of the hippocampus isolated from control animals indicated, among other thing, an increase of guanosine monophosphate (GMP) of 92.59% in the CA1 parts. Similarly, monounsaturated fatty acids (18:1, 16:1) containing sn-1 LysoPG and sn-1 LysoPE were mostly observed in CA1, while LysoPI 18:0 was detected only in the ischemia-resistant CA2–4,DG region. In the post-ischemic hippocampi, the most significant effect of the IR observed in the CA1 region was a huge accumulation of pipecolic acid (+ 2184%) in comparison to CA1 control, and + 1204% vs. post-ischemic CA2–4,DG sector. Also, a substantial reduction in citrate content upon IR treatment (by 36% and 42% in IR_CA1 vs. IR_CA2–4,DG and IR_CA 1 vs. C_CA1, respectively) was observed. All significantly altered metabolites identified are presented in Table 1.

**Table 1:** Significant metabolites discriminating studied gerbil hippocampal regions

Name	C_CA1 vs. C_CA2–4,DG		IR_CA1 vs. IR_CA2–4,DG		IR_CA1 vs. C_CA1		IR_CA2–4,DG vs. C_CA2–4,DG	
	Change	VIP	Change	VIP	Change	VIP	Change	VIP
Piperidine	+ 17.24	0.43	− 22.14	0.45	+ 42.49	*1.48*	+ 114.56	*1.58*
Hydroxypyridine	− 4.34	*1.51*	+ 24.08	*1.42*	+ 3.70	*1.16*	− 20.06	*1.41*
Indole	*	*	+ 36.59	0.48	+ 197.30	*1.75*	IR_CA2–4,DG only	*4.12*
Taurine (S)	− 26.23	*1.80*	− 29.58	0.55	− 14.68	0.33	− 10.62	*1.77*
Pipecolic acid (S)	+ 6.69	0.11	+ 1204.05	*1.11*	+ 2184.54	*1.11*	+ 86.91	0.59
Leucine (S)	+ 30.07	0.19	− 18.53	0.39	+ 32.32	*1.22*	+ 111.27	*1.57*
Histidine (S)	+ 65.68	0.49	+ 160.97	0.71	+ 360.98	*1.54*	+ 192.65	*1.72*
Phenylalanine (S)	+ 16.88	*1.48*	+ 4.40	0.15	+ 103.72	*1.02*	+ 128.09	*1.69*
3-Methylhistidine (S)	+ 111.24	0.38	+ 57.58	0.44	+ 22.75	*1.23*	+ 64.55	*1.49*
Citric acid (S)	+ 23.11	0.41	− 32.42	*2.10*	− 45.87	*1.85*	− 1.40	0.37
Tryptophan (S)	+ 9.08	0.24	+ 6.74	0.13	+ 136.91	0.98	+ 142.10	*1.08*
Carnosine	+ 23.83	0.34	+ 134.36	*1.61*	+ 86.11	*1.38*	− 1.67	0.12
Adenosine (S)	− 24.97	0.41	− 23.69	*1.60*	+ 39.67	0.45	+ 37.33	*1.38*
Acetylaspartylglutamic acid	− 30.20	0.55	+ 6.26	*1.05*	+ 6.99	*1.83*	− 29.72	*1.63*
Guanosine monophosphate	+ 92.59	*1.86*	+ 8.52	0.31	+ 3.22	0.08	+ 83.19	*2.22*
Stearoylcarnitine (S)	− 3.23	0.18	− 50.77	0.91	+ 51.59	0.46	+ 197.97	*1.23*
Arachidonoylcarnitine	− 12.30	0.28	+ 29.36	0.44	− 40.30	0.71	− 59.53	*1.11*
Eicoseneoylcarnitine	− 11.15	0.31	− 39.74	*1.63*	− 9.91	*1.45*	+ 32.82	0.53
LysoPE 18:1 sn-1	+ 48.19	0.54	+ 107.34	*1.76*	− 18.62	0.24	− 41.83	*1.79*
LysoPE 20:4 sn-1	− 21.01	0.28	− 72.07	*1.95*	− 80.80	*1.99*	− 45.71	0.56
LysoPG 18:1 sn-1 (S)	+ 195.81	*1.61*	*	*	+ 0.62	*1.55*	*	*
LysoPE 22:6 sn-1	+ 57.55	*1.90*	*	*	*	*	+ 8.97	*1.70*
LysoPI 18:1 sn-1	+ 20.34	*1.37*	+20.07	0.49	+ 44.90	0.40	+ 45.22	*1.74*
LysoPI 18:0 sn-1 (S)	C_CA2–4,DG only	*3.29*	*	*	*	*	− 2.63	*1.51*
PE 22:6/P-16:0	− 45.58	*1.53*	− 25.19	*1.50*	− 59.58	0.23	− 70.60	0.64
PE 16:1/22:6	+ 66.81	0.62	+ 36.93	*1.25*	− 30.25	0.66	− 15.02	*1.64*
PE 16:1/16:0	+ 62.98	*1.41*	+ 46.07	*1.46*	− 44.90	0.27	− 38.52	0.75
PE 22:6/18:1	*	*	− 43.69	*1.49*	*	*	− 8.38	0.05
PC 18:1/18:1	− 7.46	*1.43*	− 3.44	*1.08*	+ 33.25	0.21	+ 27.69	*1.59*
PE 20:4/22:6	+ 72.15	*1.26*	− 23.61	0.45	− 38.35	*1.32*	+ 38.93	0.61

*C_CA1*, control CA1 region of the hippocampal region; *C_CA2–4,DG*, control CA2–4,DG hippocampal regions; *IR_CA1*, CA1 hippocampal region after ischemia-reperfusion injury; *IR_CA2–4,DG*, CA2–4,DG hippocampal region after ischemia-reperfusion injury. The direction of change indicates an increased (+) or decreased (−) abundance of metabolites in the given comparison, e.g., C_CA1 vs. C_CA2–4,DG—(+)/(−) means an increased/decreased abundance of metabolites in the C_CA1 group in comparison to the C_CA2–4,DG group. *Did not pass the filtering procedures described in the “Materials and Methods” section. (S): The identities of these metabolites were confirmed by the LC-MS/MS analysis of the standards

### Metabolic Pathway Analysis

**Fig. 3:**
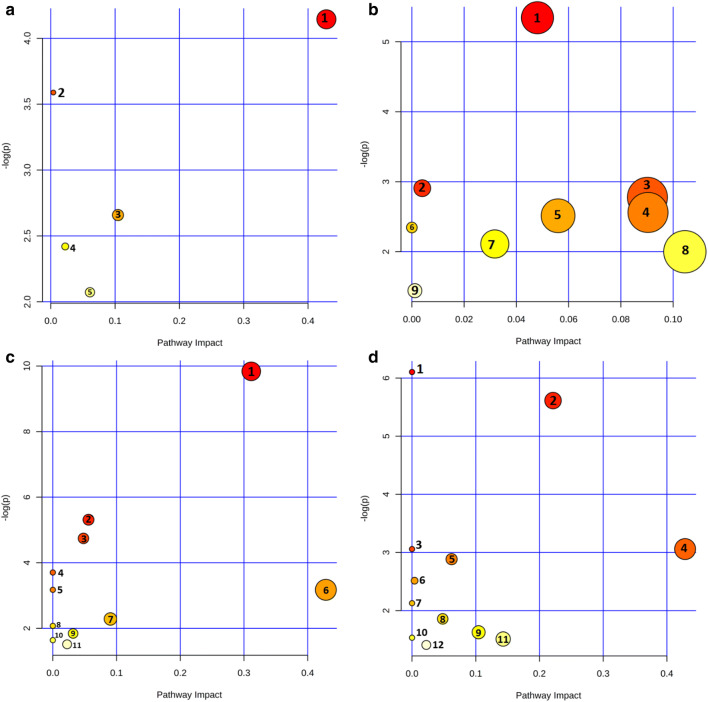
A summary of pathway analysis for metabolites significant in performed comparisons. Panel **a**: The results of pathway analysis for metabolites significant in the comparison C_CA1 vs. C_CA2–4,DG. 1, taurine and hypotaurine metabolisms; 2, glycosylphosphatidylinositol (GPI)-anchor biosynthesis; 3, glycerophospholipid metabolism; 4, primary bile acid biosynthesis; 5, purine metabolism. Panel **b**: The results of pathway analysis for metabolites significant in the comparison IR_CA1 vs. IR_CA2–4,DG. 1, alanine, aspartate, and glutamate metabolisms; 2, glycosylphosphatidylinositol (GPI)-anchor biosynthesis; 3, histidine metabolism; 4, citric acid cycle; 5, alanine metabolism; 6, lysine degradation; 7, glyoxylate and dicarboxylate metabolism; 8, glycerophospholipid metabolism; 9, purine metabolism. Panel **c**: The results of pathway analysis for metabolites significant in the comparison C_CA1 vs. IR_CA1. 1, histidine metabolism; 2, alanine metabolism; 3, alanine, aspartate, and glutamate metabolisms; 4, aminoacyl-tRNA biosynthesis; 5, valine, leucine, and isoleucine biosynthesis; 6, taurine and hypotaurine metabolism; 7, citric acid cycle; 8, lysine degradation; 9, glyoxylate and dicarboxylate metabolisms; 10, valine, leucine, and isoleucine degradation; 11, primary bile acid biosynthesis. Panel **d**: The results of pathway analysis for metabolites significant in the comparison C_CA2–4,DG vs. IR_CA2–4,DG. 1, aminoacyl-tRNA biosynthesis; 2, histidine metabolism; 3, valine, leucine, and isoleucine biosynthesis; 4, taurine and hypotaurine metabolisms; 5, purine metabolism; 6, glycosylphosphatidylinositol (GPI)-anchor biosynthesis; 7, alanine metabolism; 8, alanine, aspartate, and glutamate metabolism; 9, glycerophospholipid metabolism; 10, valine, leucine, and isoleucine degradation; 11, tryptophan metabolism; 12, primary bile acid biosynthesis

The metabolites which levels were substantially influenced in the ischemia vulnerable CA1 and ischemia-resistant CA2–4,DG segments of hippocampi isolated from control and IR animals were forwarded for pathway analysis with the use of MetaboAnalyst 4.0. Four analyses were performed, one for each comparison. As shown in Fig. 3 (panel a), taurine and hypotaurine metabolism were considered key metabolisms distinguishing control CA2–4,DG from CA1. Moreover, glycerophospholipid metabolism, purine metabolism, and primary bile acid biosynthesis pathway also differentiated hippocampal sectors in the control brain. As a result of the ischemic episode and 1-h reperfusion, the tested areas turned out to be metabolically different than in the control state (Fig. 3, panel b). Glycerophospholipid metabolism, histidine metabolism, citric acid cycle, and alanine, aspartate, and glutamate metabolism were pointed as the pathways with the highest impact. The most visible changes were detected in both tested regions after IR in comparison to the control state. Figure 3 panel c shows metabolic pathways affected by ischemia and reperfusion in the CA1 region, while panel d shows the CA2–4,DG region. In the both cases, taurine and hypotaurine metabolisms, as well as histidine metabolism, were ascribed as the most perturbed pathways. In addition, considering the pathway impact, aminoacyl-tRNA biosynthesis and tryptophan metabolism were noted as the first and third, respectively, most important pathways in CA2–4,DG, while the citric acid cycle was pointed out in the CA1.

## Discussion

Experiments described in this paper were initiated by convincing morphological data indicating that the CA1 region of the hippocampus is substantially more vulnerable to post-ischemic damage than CA2–4,DG sectors of this structure. Thus, the major goal of this study was to explain the biochemical basis of these differences. To do this, three mutually non-exclusive possibilities have been tested with the use of the metabolomics approach: (i) differences in the metabolic profile observed in CA1 and CA2–4,DG of untreated animals (control) may explain the fact that CA1 is more sensitive to IR challenge, (ii) IR stress induces a specific adaptive (protective) response of CA2–4,DG but not the CA1 region of the hippocampus; (iii) IR stress selectively induces specific effects in the CA1 region which makes this part of the hippocampus more prone to IR-evoked damage.

Although detection of changes in a single metabolite content does not allow concluding about the effect of the IR on a metabolic pathway as a whole, it may indicate putative regulatory points of particularly high sensitivity to such a stress. In this study, differences in metabolic profiles between CA1 and CA2–4,DG under control conditions and upon 5 min of ischemia followed by 1-h-lasting reperfusion were considered a strategy which could give a lead to understand why some regions of the hippocampus are more vulnerable to the post-ischemic injury than others. In fact, some observations do not enable themselves to unambiguous interpretation, but because of their high reproducibility, may be used as valuable indicators of different sensitivities of hippocampal regions to IR and shed light on metabolic changes observed in the early period of reperfusion, when intracellular signal transmission that could lead to death or to cell protection is activated [27, 28].

Metabolic pathway analysis with the use of the MetaboAnalyst 4.0 tool indicates substantial differences between CA1 and CA2–4,DG regions isolated from control animals. Additionally, more visible dissimilarities appear after the IR stress. Under control conditions, there are substantial differences between studied hippocampal regions concerning level of metabolites which may be ascribed to 5 metabolic pathways, while the IR treatment increases this number to 9. Therefore, one could suggest that CA1 and CA2–4,DG regions respond differently to the IR. In control hippocampi, the metabolisms of taurine and hypotaurine have the greatest impact and this discrepancy is still visible after IR. Also, the differences in glycosylphosphatidylinositol (GPI)-anchor biosynthesis, glycerophospholipid metabolism, and purine metabolism pathways are sustained. Additionally, after IR insult, metabolism of amino acids and citric acid differentiates two examined areas. However, metabolic pathways shown in Fig. 3b seem to be less disturbed than the disturbed pathways in other panels of Fig. 3, suggesting that after IR insult, CA1 and CA2–4,DG are more metabolically similar to each other than in control conditions. We speculate that this is an attempt to recover from IR stress by both CA1 and CA2–4,DG. Keeping in mind that morphological sings of cell death are seen not earlier than 2–4 days after ischemia, we can suggest that this attempt is not sufficient in a case of CA1. It is worth checking at what time after the restoration of circulation, CA2–4,DG metabolism begins to differ fundamentally from that in CA1 to find a process and time course that determine survival because the changes observed in CA1 at 1 h after ischemia are not enough to overcome the stress.

Table 1 contains a list of metabolites which we found, fulfilling the aforementioned criteria. Some of them may be gathered to well-defined classes according to their metabolic properties and common metabolic pathways. This is especially visible for a set of diacyl and monoacyl (lyso) glycerophospholipids, which substantially differ between CA1 and CA2–4,DG in control animals not challenged by any stressful condition. It is not clear to what extent, if at all, such a distinct lipid signature of the dorsal and abdominal hippocampus reflects their vulnerability or resistance to the IR. However, similar differences which might suggest heterogeneity of membrane lipid composition between two regions of the hippocampus were observed by other authors [38]. Such an uneven distribution of phospholipids may suggest differences in permeability, stability, and electrical properties of the plasma membranes and finally the excitability of individual cells.

Metabolomic analysis of control hippocampi has revealed a much higher amount of taurine in the CA2–4,DG than in the CA1 region. Taurine is known as a trophic factor, particularly important during the central nervous system (CNS) development. It also acts as a neurotransmitter and neuromodulator while liberated upon depolarization. It has also been found that taurine protects neurons against glutamate-induced neurotoxicity and participates together with other osmolytes in a regulation of cell volume. Extracellular taurine inhibits neuronal firing through GABA and glycine receptors. However, the existence of specific taurine receptors is still not excluded [39]. Our data also shows less taurine in the control CA1 than in the control CA2–4,DG, which one can interpret as a natural feature of the ischemia-resistant abdominal part of the hippocampus. IR episode does not affect the relative taurine content in both hippocampal parts tested (its amount is partially reduced in comparison to the control), which suggests that taurine may have protective properties in the CA2–4,DG region. Considering the function of taurine as an osmolyte, this result suggests high importance of cell volume regulation for neuron’s survival. Accumulated data also shows a significant neuroprotective role of taurine against stroke pathophysiology [40].

It is commonly accepted that the detrimental effect of the short time of ischemia followed by reperfusion relies on oxidative damage to a variety of macromolecules, particularly localized in mitochondria, as these organelles are the major source of ROS in animal cells challenged by various stressors. Chen and co-workers [41] described a substantially increased citrate level in the brain cortex isolated from mice upon transient ischemia (2 h MCAO + 24 h of reperfusion). They suggested that citrate accumulation resulted from an increased ROS generation and, therefore, inhibition of citrate conversion to isocitrate by cis-aconitase. This enzyme is particularly susceptible to the ROS-evoked inactivation due to the 4Fe-4S cluster located in the activity center. Interestingly, data shown in Table 1 indicates that the IR procedure we applied differently influenced the citrate level in both regions of the hippocampus tested. In the CA2–CA4,DG, the citrate concentration was unaffected while in the CA1, it was significantly reduced. The latter observation may suggest reduced acetyl-CoA supply in CA1 and limited TCA activity. Indeed, substantially reduced level of stearoylcarnitine and eicoseneoylcarnitine in CA1 shown after the IR could indicate lowered delivery of fatty acids to mitochondria; however, this hypothetical explanation needs more experiments to be verified. It must also be emphasized that the experimental procedure used in our study was significantly different from Chen’s protocol. Five-minute-lasting ischemia results in a relatively gentle brain injury, which is visible with the use of morphologic approaches not earlier than on the 4th day of reperfusion. Moreover, no symptoms of local necrosis were observed in the ischemia-vulnerable CA1 although delayed apoptotic cell death was confirmed. So, both models are substantially different as they concern different pathological events.

A profound increase in the pipecolic acid (PA) content in CA1 and its almost negligible change in the CA2–4,DG region additionally underline the specificity and unevenness of metabolic response of hippocampi challenged by IR stress. Pipecolic acid is an intermediary product in CNS-specific lysine degradation [42]. It is suggested that PA is involved in either synaptic transmission or in its modulation at GABA synapses in (CNS) [43]. However, reports of its effects on the GABAergic system are ambiguous [39, 44]. PA was found to potentiate GABA action in rat CNS: it reduces GABA reuptake and stimulates its release—presynaptic effect [40], so it might potentially have a protective effect in the ischemic brain. This scenario seems to be less probable in view of the fact of the particularly high vulnerability of the CA1 to the IR stress. On the other hand, it may be possible that an increased level of PA in the post-ischemic CA1 region reflects pro-survival response, which is insufficient to fully protect cells against death under experimental conditions (but it may prevent more severe damage). An increased l-pipecolic acid concentration was reported in the plasma and the cerebrospinal fluid in children with pyridoxine-dependent epilepsy [45] and in the plasma samples of sleep apnea and hypopnea syndrome patients [46]. These observations indicate that pipecolic acid is worth considering during further studies on CNS damage.

*N*-Acetylaspartylglutamate (NAAG) is a small peptide present primarily in the nervous tissue [47]. There are several data showing its excitatory action on neurons [48–50] and in the brain cortex and hippocampal slices [51]. Moreover, it is suggested that the neuroexcitatory action of NAAG on spinal cord neurons is due to selective activation of NMDA receptors, but such an effect was only observed at high concentrations of this compound [52]. Recently NAAG has been described to be a full agonist of mGluR3, exhibiting a protective activity in focal cerebral ischemia and in the neonatal rat model of hypoxia-ischemia [53, 54]. Accordingly, peritoneal injection of NAAG in neonatal rat brain, which is the hypoxia-ischemia (H-I) model of birth asphyxia, reduced weight loss in the ischemic hemisphere and mitigated neuronal degeneration in the CA1 hippocampal region and cerebral cortex. NAAG reduced ROS levels in the ipsilateral hemisphere that was observed after H-I and prevented an increase in antioxidant enzyme activity in the injured hemisphere, restoring them to control levels [55]. Our results show that NAAG is mostly present in the control CA2–4,DG region of the hippocampus that might be one of the endogenous features of ischemic resistance to ischemic episodes. IR changes the relative NAAG content in the CA1 and CA2–4,DG regions, which suggests that the elevation of NAAG in the CA1 region is a rescue attempt observed early after transient ischemia. According to such an explanation, our data are in agreement with that reported by [41], where the elevation of NAAG is suggested as one of the protective mechanisms of hydrogen in mice with ischemic stroke. However, the function of the endogenous NAAG might not only rely on mGluR3 stimulation, so in CA2–4,DG, its amount is mitigated after IR in comparison to control. In general, our data suggest that there are spatial differences in the metabolism of amino acids and its metabolites in the hippocampus that regulate neuronal function, with a predominance of those which upkeep for their well-being in the CA2–4,DG region.

Metabolic pathway analysis also suggests differences in an activation of aminoacyl-tRNA biosynthesis and amino acid metabolism. Aminoacyl-tRNA biosynthesis has been noted as a more significant pathway in post-ischemic CA2–4,DG than in ischemia vulnerable CA1. Activation of tRNA aminoacylation is an important strategy impeding an essential step of protein synthesis while a dynamic regulation of mRNA translation is essential for the survival and function of neuronal cells [56–58] Moreover, it was suggested that a long-lasting suppression of protein synthesis is one of events corresponding to post-ischemic neuronal death [59]. While the stress-induced shutdown of translation is viewed as a protective response, the inability of vulnerable cells to restore protein synthesis after being exposed to a severe form of stress is a pathological process because it blocks the translation of messages coding for protective proteins required for restoration of function [60].

Based on these information and above presented data, one could conclude that the high significance of the aminoacyl t-RNA biosynthesis pathway at the very early stage of the regeneration after IR seems to be of high importance for cell survival. Perhaps this is one of the most important distinguishing features of CA1 and CA2–4,DG. Following this line of thought, an activation of protein synthesis might be closely related with the neuronal protection showed in permanent middle cerebral artery occlusion (pMCAo) model of rats, where pathways of aminoacyl t-RNA synthesis and amino acid metabolisms were modulated by a traditional Chinese herbal formula and positively influenced cerebral energy metabolism [61].

There are several limitations in this study. We do not address any causative or correlative interactions between different metabolites. The role of each metabolite for IR pathology is suggested on the basis of available literature. Moreover, we do not focus on specific cell types which are present in studied samples while morphological changes are seen specifically in CA1 neurons upon prolonged reperfusion. Based on genomic approaches, it was suggested that neuronal factors dominate the selective vulnerability of CA1 [15]. But in many cases, an interaction between neurons and astrocytes is required to build the intact pathways, as for example, it was shown for taurine synthesis pathway [62] and glutamine glutamate cycle [63, 64]. Shown here, the hippocampal metabolic pattern suggests that ischemia-resistant part metabolically differs from ischemia-vulnerable CA1 which is not provided with stress survival mechanisms. Nevertheless, after ischemic insult, in very early reperfusion (1 h), the pathway analysis suggests the intrinsic attempt to save these neurons. Based on this assumption, we suggest that increasing the pro-survival cellular efforts might be a possible way to save CA1 neurons. This opens the question about the length of metabolic window which could allow using pharmacological approaches to prevent stroke-related complications. Other studies have shown an increased metabolic susceptibility of the brain after 24 h of reperfusion in much more severe model of rat cardiac arrest [25].

It is worth to point out that the number of animals used in this study is low, but it reflects EU regulations concerning 3Rs principles (Replacement, Reduction, and Refinement). In this light, in some cases, differences between samples do not reach statistical significance level adopted in these studies but each metabolite significantly discriminates samples at least in one comparison, what can point out specific metabolite and pathway which deserve more systematic studies.

## Conclusions

Untargeted metabolomics has appeared as a convenient tool to discriminate between two parts of the hippocampus characterized by distinct sensitivities towards transient ischemia-reperfusion episode. Thanks to it we were able to observe metabolic alterations of both parts in control and ischemia-reperfusion conditions, which suggested a probable source of various responses of CA1 and CA2–4,DG to IR and indicated new directions of future research.

## Supplementary information

ESM 1 (PDF 722 kb)
