# Supplementary material for: Hippocampal Sector–Specific Metabolic Profiles Reflect Endogenous Strategy for Ischemia-Reperfusion Insult Resistance
Source: Mol Neurobiol. 2020 Nov 22;58(4):1621–33. doi: 10.1007/s12035-020-02208-6 (PMC7932963; doi:10.1007/s12035-020-02208-6)
Supplement: Supplementary file 1 — (PDF 722 kb) [file 12035_2020_2208_MOESM1_ESM.pdf]

Supplementary material to: Hippocampal sector-specific metabolic profiles reflect endogenous strategy for ischemia-reperfusion insult resistance

Olga Krupska[1,3,a], Tomasz Kowalczyk[2,a], Małgorzata Beręsewicz-Haller[1], Paulina Samczuk[2], Karolina Pietrowska[2], Krzysztof Zabłocki[3], Adam Kretowski[2], Michal Ciborowski[2]* and Barbara Zabłocka[1]*

[1]Molecular Biology Unit, Mossakowski Medical Research Centre PAS Warsaw, Poland; okrupska@imdik.pan.pl; mberesewicz@imdik.pan.pl; bzablocka@imdik.pan.pl

[2]Metabolomics Laboratory, Clinical Research Centre, Medical University of Bialystok, Poland; tomasz.kowalczyk@umb.edu.pl; paulina.samczuk@gmail.com; adamkretowski@wp.pl; michal.ciborowski@umb.edu.pl; karolina.pietrowska@umb.edu.pl

[3]Laboratory of Cellular Metabolism, Department of Biochemistry, Nencki Institute of Experimental Biology, Warsaw, Poland, k.zablocki@nencki.edu.pl; o.krupska@nencki.edu.pl

* Correspondence: bzablocka@imdik.pan.pl; Tel.: (48) 22 60 86 486 (BZ); michal.ciborowski@umb.edu.pl; Tel.: (48) 85 831 81 51 (MC)

[a] equal contribution

Figure S1. PCA plots showing clustering of the QC samples.

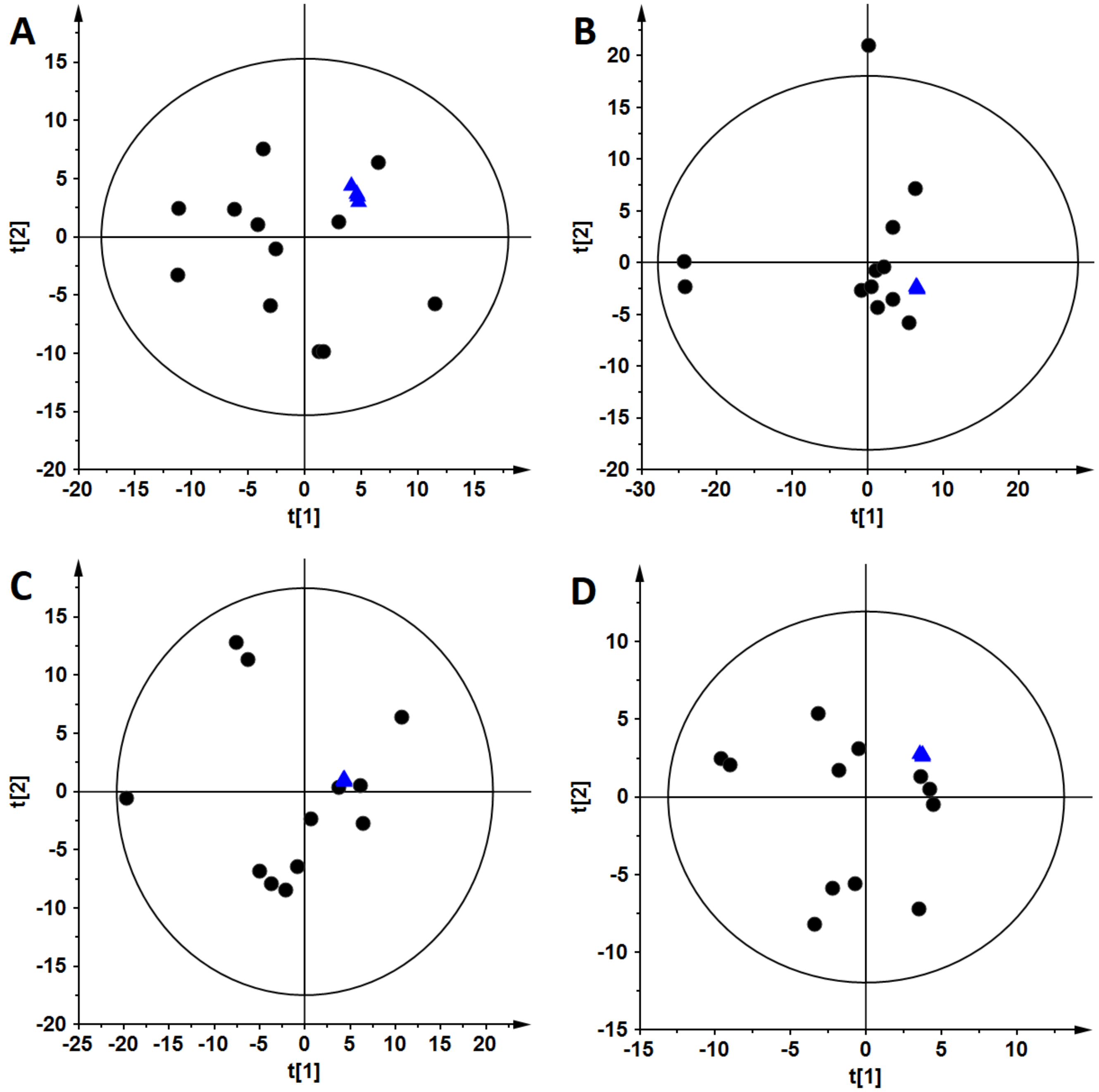

All data were log transformed and Ctr scaled. Panel A – RP(+) data, R2=0.555; Panel B – RP(-), R2=0.418; Panel C – HILIC(+), R2=0.487; Panel D – HILIC(-), R2=0.355. ● - biological samples, ▲ - QC samples.

Figure S2. Partial least square discriminant analysis plots showing discrimination between the studied hippocampal regions based on RP(-) data.

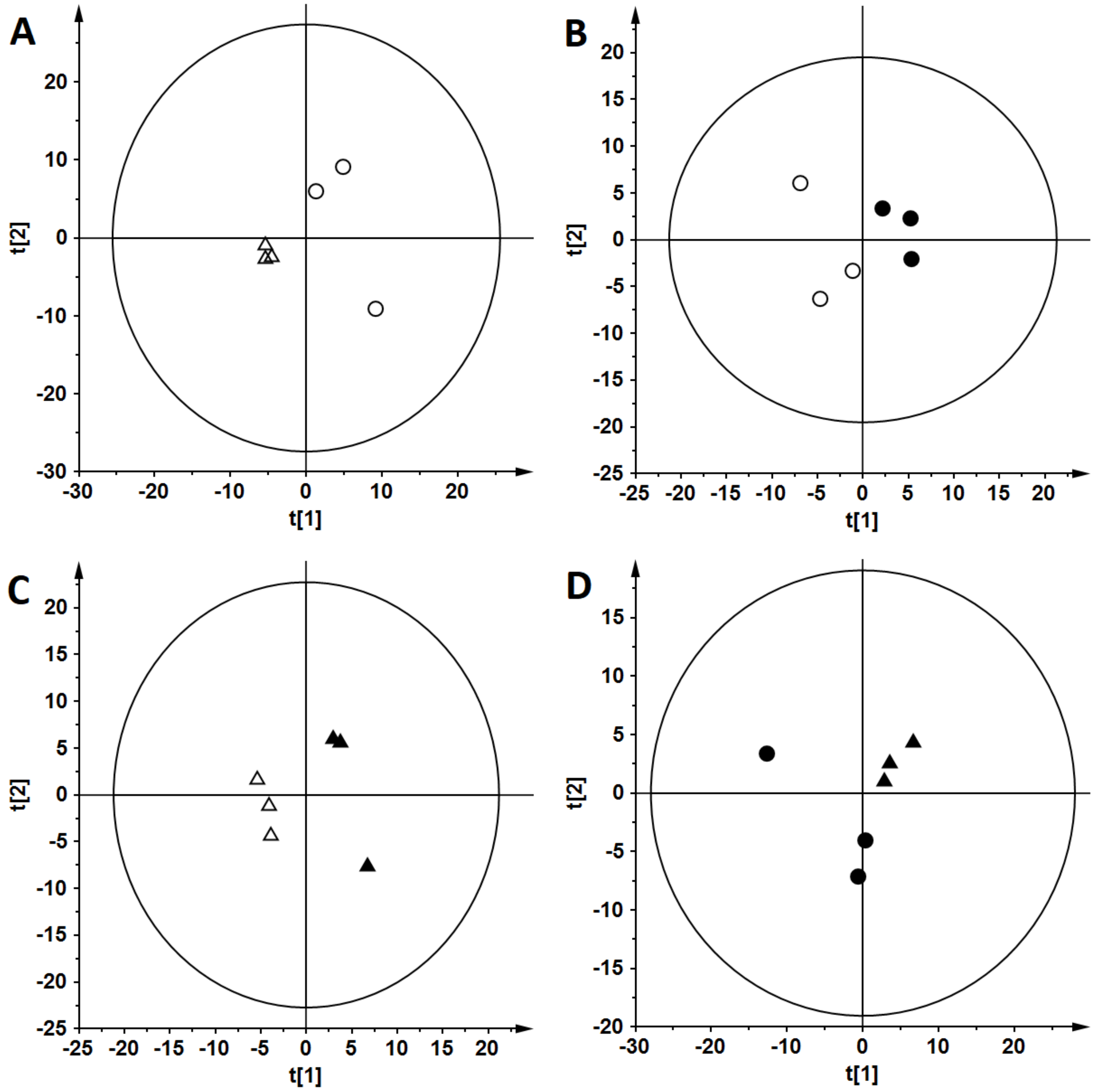

Data were log transformed and Par scaled. ○ - C_CA1; △ - C_CA2-4,DG; ● - IR_CA1; ▲ - IR_CA2-4,DG. Panel A: C_CA1 vs C_CA2-4,DG; R2=0.946, Q2=0.533. Panel B: C_CA1 vs IR_CA1; R2=0.979, Q2=0.390. Panel C: C_CA2-4,DG vs IR_CA2-4,DG; R2=0.996, Q2=0.824. Panel D: IR_CA1 vs IR_CA2-4,DG; R2=0.883, Q2=0.439.

Figure S3. Partial least square discriminant analysis plots showing discrimination between the studied hippocampal regions based on HILIC(+) data.

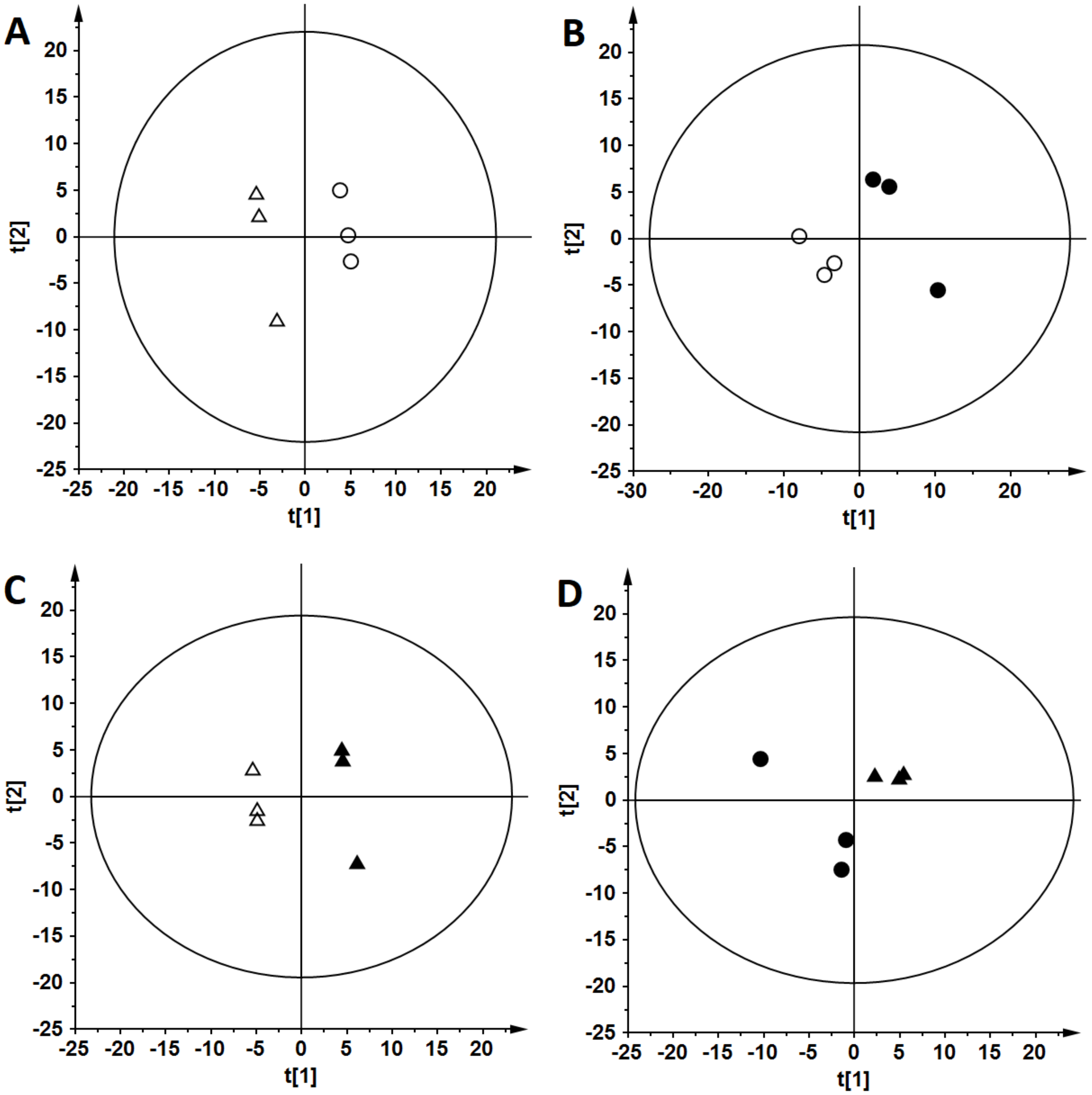

Data were log transformed and Par scaled. ○ - C_CA1; △ - C_CA2-4,DG; ● - IR_CA1; ▲ - IR_CA2-4,DG. Panel A: C_CA1 vs C_CA2-4,DG; R2=1.000, Q2=0.781. Panel B: C_CA1 vs IR_CA1; R2=0.981, Q2=0.813. Panel C: C_CA2-4,DG vs IR_CA2-4,DG; R2=1.000, Q2=0.883. Panel D: IR_CA1 vs IR_CA2-4,DG; R2=0.956, Q2=0.650.

Figure S4. Partial least square discriminant analysis plots showing discrimination between the studied hippocampal regions based on HILIC(-) data.

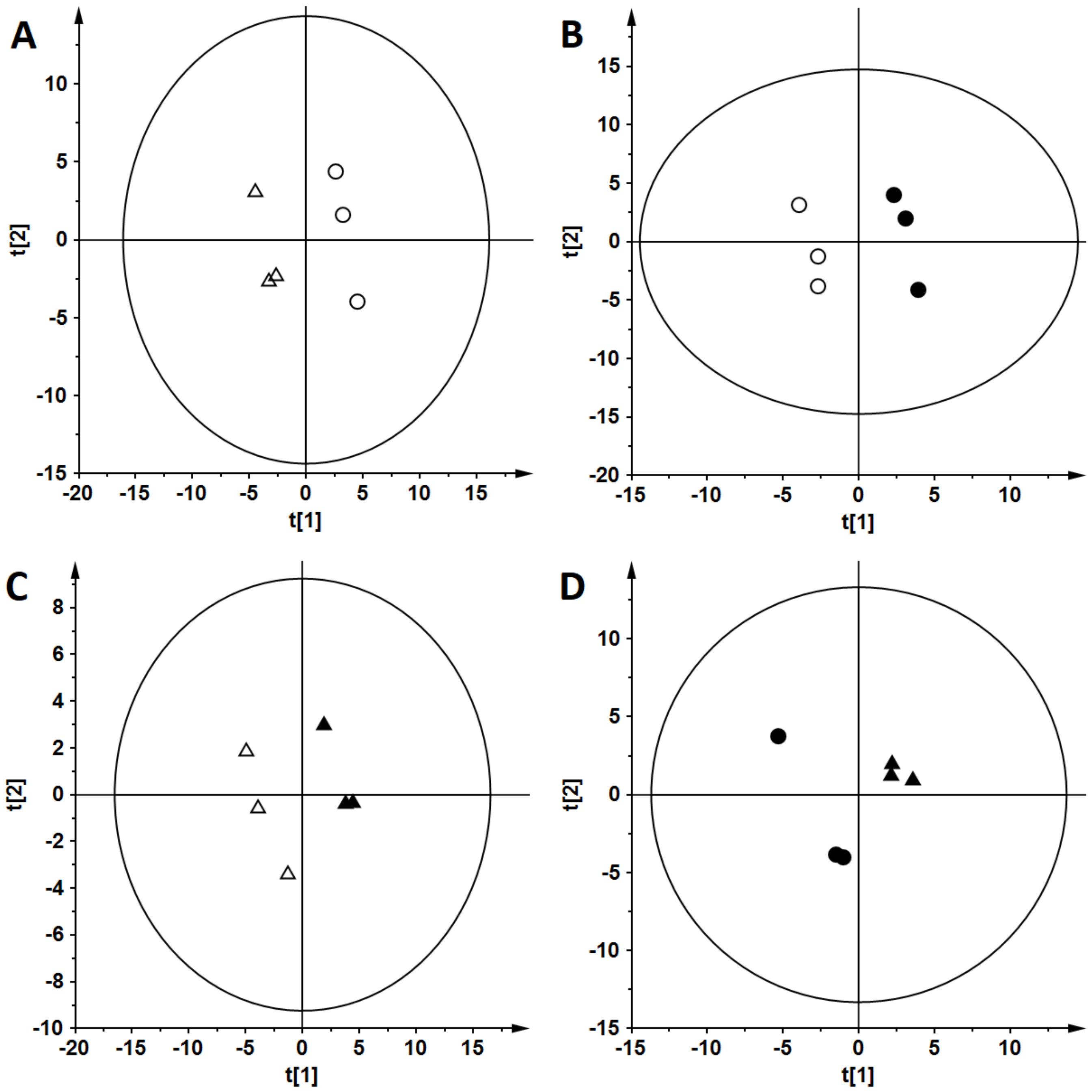

Data were log transformed and Par scaled. ○ - C_CA1; △ - C_CA2-4,DG; ● - IR_CA1; ▲ - IR_CA2-4,DG. Panel A: C_CA1 vs C_CA2-4,DG; R2=0.997, Q2=0.889. Panel B: C_CA1 vs IR_CA1; R2=0.997, Q2=0.763. Panel C: C_CA2-4,DG vs IR_CA2-4,DG; R2=0.995, Q2=0.595. Panel D: IR_CA1 vs IR_CA2-4,DG; R2=0.986, Q2=0.710.

Table S1.

Name	Experimental neutral mass [Da]	RT [min]	Error [ppm]	Experimental mass [Da]	Fragments	Adduct	Chromatography (ion mode)
Piperidine	85.08919	3.97	0	86.0957	41.0381, 42.0326, 43.0532, 44.0490, 56.0487, 57.0571, 58.0632, 69.0694, 70.0628	M+H	HILIC (pos)
Hydroxypyridine	95.0373	3.28	2	96.0437	51.0229, 78.0325	M+H	HILIC (pos)
Indole	117.0577	0.64	1	118.0843	91.0527	M+H	C8 (pos)
Taurine	125.015	0.59	3	126.0221	108.0107	M+H	C8 (pos)
Pipecolic acid	129.0795	6.67	4	130.1590	84.0810	M+H	HILIC (pos)
Leucine	131.0951	3.95	4	132.1016	44.0499, 69.0707, 86.0966	M+H	HILIC (pos)
Histidine	155.0702	6.42	5	156.0766	83.0606, 95.0605, 110.0714	M+H	HILIC (pos)
Phenylalanine	165.0791	3.70	1	164.0723	72.0085, 103.0545, 147.0443	M+H	HILIC (neg)
3-Methylhistidine	169.086	7.58	5	170.0929	96.0686, 109.0754, 124.0863	M+H	HILIC (pos)
Citric acid	192.0267	2.66	2	191.0192	43.0199, 57.0349, 71.0133, 85.0292, 87.0087, 111.0084, 129.0194	M-H	HILIC (neg)
Tryptophan	204.0905	0.65	3	205.0978	118.0636, 146.0595, 59.0922, 188.0701	M+H	C8 (pos)
Carnosine	226.108	7.53	6	227.1127	95.0611, 110.0713, 122.0712, 138.0650, 156.0766, 164.0822, 181.1076, 198.0855, 210.0871	M+H	HILIC (pos)
Adenosine	267.098	1.59	5	268.1588	136.0617	M+H	HILIC (pos)
Acetylaspartylglutamic acid	304.0909	0.64	1	303.0545	58.0302, 96.0086, 102.0559, 128.0351,146.0456, 155.0453, 182.0455, 226.0358, 285.0747	M-H	C8 (neg)
Guanosine monophosphate	363.0579	0.62	0	362.0499	78.9591, 96.9686, 150.0405, 211.0002	M-H	C8 (neg)
Stearoylcarnitine	427.3669	7.91	2	428.3729	60.0808, 85.0282, 144.10.11, 267.2670, 369.2290	M+H	C8 (pos)
Arachidonoylcarnitine	447.3351	6.70	1	448.3425	60.0813, 85.0281, 144.1022	M+H	C8 (pos)
Eicoseneoylcarnitine	453.3814	8.15	1	454.3897	60.0813, 85.0285, 144.1024, 297.2019	M+H	C8 (pos)
Lyso PE 18:1 sn-1	479.3014	7.82	0	480.3443	339.2885, 465.2962	M+H	C8 (pos)

Lyso PE 20:4 sn-1	501.2859	7.15	1	502.2893	361.2735, 459.2461, 484.2753	M+H	C8 (pos)
Lyso PG 18:1 sn-1	510.2947	9.48	2	509.2893	152.9950, 245.0397, 281.2473	M-H	C8 (neg)
Lyso PE 22:6 sn-1	525.2861	7.12	1	526.2934	385.2735	M+H	C8 (pos)
Lyso PI 18:1 sn-1	598.3112	8.79	1	597.3041	78.9582, 152.9945, 241.0111, 281.2474, 315.0472	M-H	C8 (neg)
Lyso PI 18:0 sn-1	600.3266	10.26	1	599.3199	78.9587, 152.9950, 241.0108, 283.2632, 315.0469	M-H	C8 (neg)
PE 22:6/P-16:0	747.52	14.85	0	746.5133	196.0383, 283.2440, 327.2334, 436.2832	M-H	C8 (neg)
PE 16:1/22:6	761.5004	13.34	1	760.4887	140.0099, 196.0385, 253.2150, 327.2312, 450.2606	M-H	C8 (neg)
PE 16:1/16:0	777.5538	13.41	2	776.5515	168.0435, 253.2178, 255.2335, 480.3090, 716.5235	M+FA-H	C8 (neg)
PE 22:6/18:1	789.5306	14.47	0	788.5232	140.0118, 196.0370, 281.2485, 327.2324, 478.2927	M-H	C8 (neg)
PC 18:1/18:1	831.5989	13.75	0	830.5917	168.0400, 224.0600, 281.2488, 770.5705	M+FA-H	C8 (neg)
PE 20:4/22:6	879.5058	13.39	4	879.5058	140.0103, 196.0370, 303.2329, 327.2323, 500.2762, 810.5075	M-H+HCOONa	C8 (neg)
